# Draft Genomes of Halophilic *Chromohalobacter* and *Halomonas* Strains Isolated from Brines of The Carpathian Foreland, Poland

**DOI:** 10.7150/jgen.80829

**Published:** 2023-03-21

**Authors:** Jakub Lach, Dominik Strapagiel, Agnieszka Matera-Witkiewicz, Paweł Stączek

**Affiliations:** 1 Department of Molecular Microbiology, Faculty of Biology and Environmental Protection, University of Lodz, Lodz, Poland; 2 Biobank Lab, Department of Oncobiology and Epigenetics, Faculty of Biology and Environmental Protection, University of Lodz. Lodz, Poland; 3 Screening of Biological Activity Assays and Collection of Biological Material Laboratory, Wroclaw Medical University Biobank, Faculty of Pharmacy, Wroclaw Medical University, Wroclaw, Poland

## Abstract

*Chromohalobacter* and *Halomonas* are genera of bacterial microorganisms belonging to the group of halophiles. They are characterized by high diversity and the ability to produce bioproducts of biotechnological importance, such as ectoine, biosurfactants and carotenoids. Here, we report three draft genomes of *Chromohalobacter* and two draft genomes of *Halomonas* isolated from brines. The length of the genomes ranged from 3.6 Mbp to 3.8 Mbp, and GC content was in the 60.11%-66.46% range. None of the analysed genomes has been assigned to any previously known species of the genus *Chromohalobacter* or *Halomonas*. Phylogenetic analysis revealed that *Chromohalobacter* 296-RDG and *Chromohalobacter* 48-RD10 belonged to the same species, and *Chromohalobacter* 11-W is more distantly related to the other two analysed strains than to *Chromohalobacter canadensis*.* Halomonas* strains 11-S5 and 25-S5 were clustered together and located close to *Halomonas ventosae.* Functional analysis revealed BGCs related to ectoine production in all genomes analysed. This study increases our overall understanding of halophilic bacteria and is also consistent with the notion that members of this group have significant potential as useful natural product producers.

## Introduction

Hypersaline ecosystems have been widely explored mainly because of their unique biodiversity and biotechnological potential. It is related to the adaptation of halophiles to the conditions of high osmotic pressure, limited availability of energy resources and other unfavourable environmental circumstances 1. Novel halophilic microorganisms have been isolated in recent years from habitats such as saline lakes, salt mines, saline soils or fermented foods 2-6. A great variety of saline environments related to factors other than salinity itself, such as pH, temperature or the availability of nutrients, results in a considerable diversity of inhabiting microorganisms, both in terms of genetics and metabolism.

*Halomonadaceae* is a family of halophilic *Gammaproteobacteria,* including *Chromohalobacter*, *Halomonas* and 12 other genera 7,8*.* Currently, *Chromohalobacter* genus includes eight validly published species isolated from salterns, seas and food products. The process of validating the publication of new species of prokaryotes is related to the fulfilment of all of the requirements set out in the “International Code of Nomenclature of Prokaryotes” 9. Moreover, the new species *Chromohalobacter moromii* sp. nov. isolated from lupine-based moromi fermentation has been described and is pending validation 6. Microorganisms belonging to the genus *Chromohalobacter*, for example, *Chromohalobacter salexigens*, have been identified as producers of biotechnologically valuable compounds and, due to their genomic characteristics, could become a useful metabolic engineering tool for the overproduction of ectoines 10.

On the other hand, the *Halomonas* genus includes 117 validly published species and new species like *Halomonas alkalisoli* sp. nov are waiting for validation 2. Most of these species are widely distributed in saline habitats, such as salt lakes, marine environments, and saline soils 11,12. *Halomonas* strains exhibit high metabolic and physiological versatility, thus a wide range of bioproducts, such as ectoine, glycine betaine and polyhydroxyalkanoates (PHA) can be produced 12,13.

This study presents the characteristics of five genomes of microorganisms isolated from brines, sources of which are located in the southern part of the Carpathian Foreland in Poland near Kraków city. Three genome sequences of *Chromohalobacter sp.* and two draft genome sequences of *Halomonas sp* are reported*.*

## Materials and methods

Three strains of *Chromohalobacter sp.* and two strains of *Halomonas sp.* were isolated from brines. *Chromohalobacter* 11-W was isolated from the borehole of the former Barycz mining area (49^◦^59'05” N 20^◦^00'52” E), *Chromohalobacter* 296-RDG and *Chromohalobacter* 48-RD10 were isolated from the Bochnia Salt Mine (49°58′09″N 20°25′03″E) and *Halomonas* strains 11-S5 and 25-S5 were isolated from the brine source in Łapczyca (49°57'30"N 20°21'41"E). Strains were cultured in 28°C on plates containing halobacteria medium (DSMZ 372) with 15% NaCl addition for strains *Chromohalobacter* 11-W, *Chromohalobacter* 296-RDG and *Halomonas* 25-S5. Rest of the strains were cultured in the same conditions except NaCl concentration which was changed to 20%. Medium was solidified with 2% agar. For genomic DNA extraction QIAamp DNA Mini Kit (Qiagen, Hilden, Germany) has been used.

Paired-end libraries were prepared from 1 ng of high-quality genomic DNA with the Nextera XT DNA sample preparation kit according to the manufacturer's instructions (Illumina Inc., San Diego, USA). The libraries were sequenced using a NextSeq 500 instrument (Illumina, San Diego, USA) at a read length of 2 × 150 bp in Biobank Lab, University of Lodz. The quality of reads was checked using FastQC 14. Furthermore, adaptors and low-quality sequences were removed from the reads with trim galore v. 0.6.4 on default parameters 15. *De novo* assembly was performed with SPAdes v3.15.0 16. Contigs with coverage lower than 2, or lengths lower than 500 bp, were removed from the assembly. Contamination and completeness of assemblies were calculated using CheckM based on a reference database of marker genes 17. Overall statistics of assemblies quality parameters were tested using Quast 18. Genomes annotation was performed using Prokka v.1.14.0 19 and the eggNOG-mapper website v.5.0.0 20. Biosynthetic gene clusters (BGCs) prediction was conducted with antiSMASH v.6.0.1 21. Taxonomic annotation of genomes was performed with gtdb-tk version 1.5.1 22. Phylogenomic analysis was accomplished on Type (Strain) Genome Server (TYGS) 23, and the whole-genome sequence-based phylogenetic tree was visualized using iTOL 24. For TYGS analysis, 27 reference strain genomes were used. They are available in the NCBI database under accessions: NC_007963, NZ_BNAE01000000, NZ_CAAHFN010000000, NZ_CABVOU010000000, NZ_CP018139, NZ_FNIV01000000, NZ_FOBC01000000, NZ_FPAQ01000000, NZ_JACHXQ010000000, NZ_JACHXR010000000, NZ_JACHZF010000000, NZ_JAGXFD010000000, NZ_JAKGAJ010000000, NZ_JAKGAK010000000, NZ_JAKGAL010000000, NZ_JAKGAM010000000, NZ_JAKGAN010000000, NZ_PYVX01000000, NZ_PZJV01000000, NZ_QPIJ01000000, NZ_RXNS01000000, NZ_SDMO01000000, NZ_SNZJ01000000, NZ_SOBR01000000, NZ_VBUI01000000, NZ_WUTS01000000, NZ_WUTT01000000. Phylogenetic tree for 16S rRNA gene sequences was prepared in MEGA X software 25.

All sequencing data are publicly available from the National Institutes of Health under BioProject accessions PRJNA899688, PRJNA899690, PRJNA899692, PRJNA899693, PRJNA899694.

## Results and discussion

Each draft genome was composed of between 38 and 321 contigs, with genome sizes ranging 3.6-3.9 Mbps. The overall genome completeness was estimated at between 98.71-99.86%, with contamination in the range of 0.54-8.42 and GC content in the 60.11-66.46% range. The summary is presented in Table 1.

**Figure 1:**
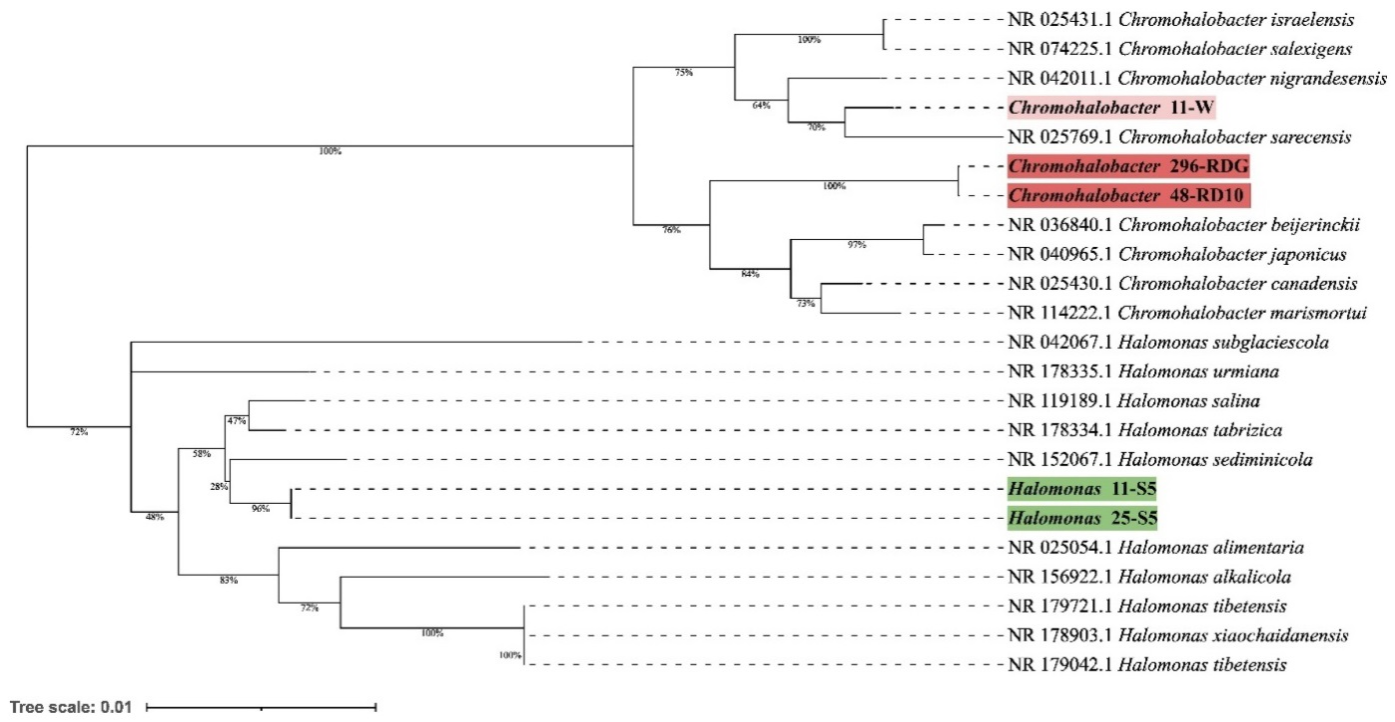
Phylogenetic tree prepared based on 16S rRNA gene sequences analysis using the Neighbor-Joining method and showing the relationships between analysed strains and other *Chromohalobacter* and *Halomonas* strains. The evolutionary distances were computed using the Kimura 2-parameter method.

**Figure 2:**
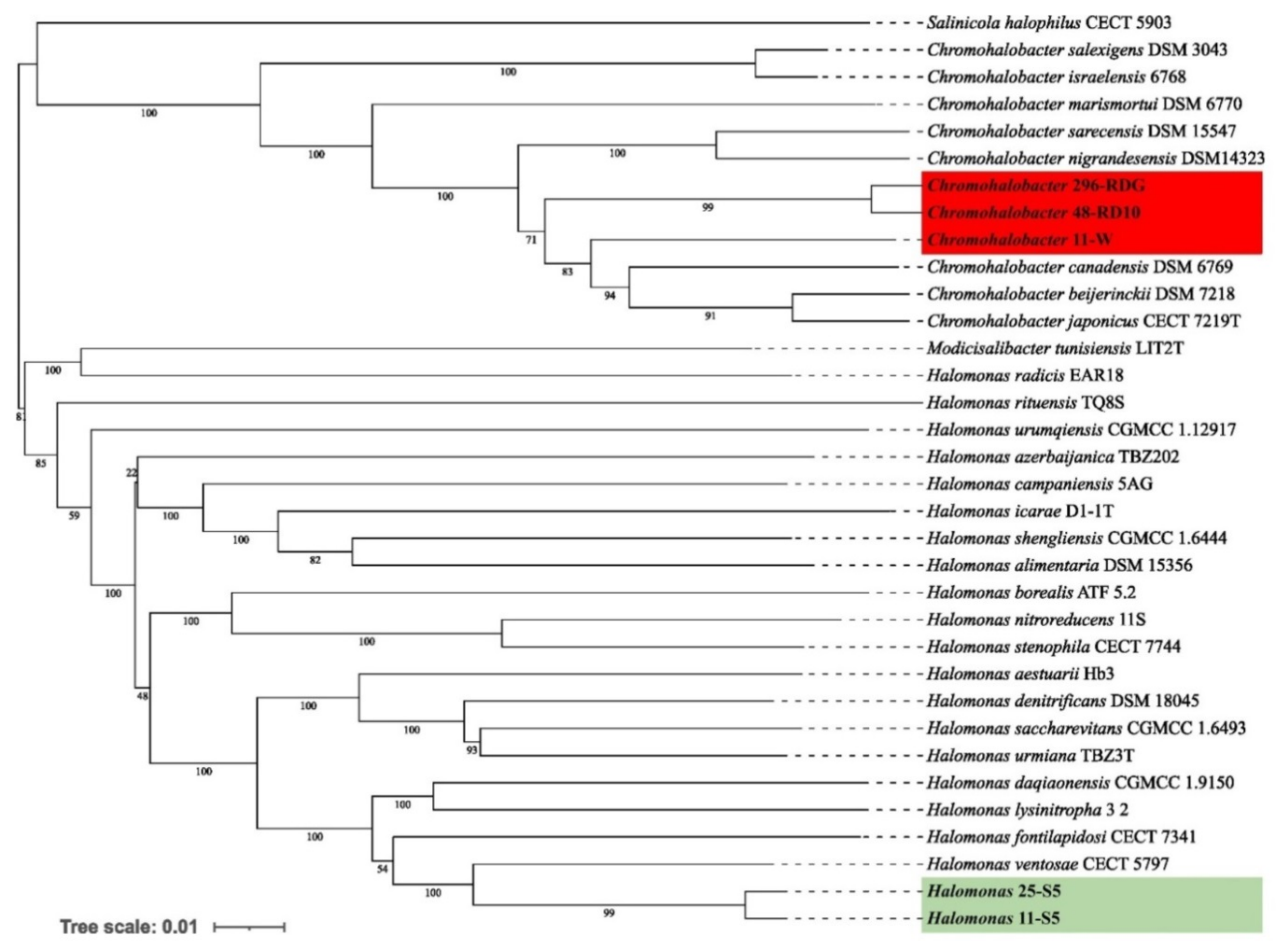
Whole-genome sequence-based phylogenetic tree build using Genome BLAST Distance Phylogeny approach (GBDP) on the Type (Strain) Genome Server (TYGS) platform showing the phylogenetic relationships between analysed Bacteria strains and other close related to them species.

Preliminary taxonomic annotation of genomes using gtdb-tk, assigned isolates 11-W, 296-RDG, and 48-RD10 to *Chromohalobacter* genus, and isolates 11-S5, 25-S5 to *Halomonas* genus. The assignment to the species level was impossible because of too high differences in genome sequences between the analysed strains and the previously described genomes available in the databases. A phylogenetic analysis was performed in order to deepen knowledge about the relationship between the analysed isolates and other species. A phylogenetic tree, based on the 16S rRNA gene sequences was built. The resulting tree confirmed that the isolates 11-W, 296-RDG, and 48-RD10 fell within a cluster comprising members of the genus *Chromohalobacter* and the strains 11-S5, 25-S5 fell within a cluster including members of the genus *Halomonas* (Figure 1)*.* However, in both cases, the analysed strains were separated from the other species included in the analysis. The closest species for *Chromohalobacter* strains 296-RDG and 48-RD10 was *Chromohalobacter canadensis*, and for strain 11-W, it was *Chromohalobacter sarecensis*. In the case of *Halomonas* strains, the closest taxon was *Halomonas sediminicola.* This initial phylogenetic analysis, using a comparison of 16S rRNA gene sequences, was then deepened through the construction of a further, whole-genome sequence-based phylogenetic tree build using Genome BLAST Distance Phylogeny approach (GBDP) created on the TYGS platform (Figure 2). The obtained phylogenetic tree confirmed the observations made at an earlier stage. On the genome-wide scale, it was noticed that *Chromohalobacter* 11-W is more distant from the other two analysed strains than from *Chromohalobacter canadensis.* That may suggest genomes assignment to two different species of *Chromohalobacter.* This observation was confirmed by digital DNA-DNA hybridization (dDDH) evaluation, where the similarity between *Chromohalobacter* 296-RDG and 48-RD10 was 87.5% (d4 method), between 11-W and 296-RDG it was 42.7 % and between 11-W and 48-RD10 it reached 42.9%*. Halomonas* strains 11-S5 and 25-S5 were clustered together on the whole-genome tree. Allocation to the same species was confirmed by dDDH which was 89.7%. The closest related species to the analysed strains was *Halomonas ventosae*.

Functional annotation of genomes revealed that they all contained numerous genes involved in the biosynthesis of secondary metabolites (Table 2). However, both *Halomonas* strains, 11-S5 and 25-S5, had a higher number of genes belonging to this category (99 and 101 genes, respectively) than isolates belonging to the genus *Chromohalobacter*, which consisted of 69-89 such genes, depending on the strain. This observation is consistent with previous reports that *Halomonas* and *Chromohalobacter* have a high diversity of biosynthetic processes 10,12,13 Based on these results, the annotation of BGCs with antiSMASH was performed. Interestingly, as a result, more BGCs were identified in *Chromohalobacter* strains than *Halomonas*, despite a smaller number of genes associated with processes identified during the analysis. Moreover, BGCs related to ectoine production have been identified in all genomes. Most likely, it is related to the adaptation of the studied microorganisms to conditions of high salinity. Ectoine produced by the analysed strains is one of the most important compatible solutes that protects the cell against high osmotic pressure 26. In the 11-W, 296-RDG, 48-RD10, and 11-S5 strains, a complete operon *ectABC* was identified. In the 25-S5 strain, only the *ectC* gene, essential for ectoine production, was identified. In *Halomonas* isolates, BGCs associated with ectoine production were the only BGCs identified in the genomes. In *Chromohalobacter* strains, BGCs related to the production of siderophores, redox-cofactors, and arylopolyenes, were also identified. Table 3 summarizes the information on the identified BGCs in each of the strains.

To summarize, the draft genomes of three *Chromohalobacter* strains and two *Halomonas* strains expand the genomic representation in the tree of life. The strains analysed were isolated from the hitherto unexplored saline environment, which allows a deeper understanding of their biodiversity.

## Figures and Tables

**Table 1:** Genome features

	*Chromohalobacter* 11-W	*Chromohalobacter* 296-RDG	*Chromohalobacter* 48-RD10	*Halomonas* 11-S5	*Halomonas* 25-S5
Genome length (bp)	3 660 465	3 763 726	3 785 492	3 634 207	3 872 356
Number of contigs	38	321	222	65	101
Largest contig (bp)	607 581	256 035	627 765	320 470	407 008
GC content (%)	60.83	60.11	60.3	66.46	65.9
N50 (bp)	318 640	58 943	228 913	105 985	122 550
Number of CDSs	3 360	3 576	3 572	3 357	3 551
Number of rRNAs	6	3	3	7	5
Number of tRNAs	63	63	65	60	58
Number of repeat regions	3	2	3	1	2
Completeness (%)	98.71	98.71	99.57	99.86	99.86
Contamination (%)	0.86	0.86	8.42	0.68	0.54

**Table 2:** eggNOG categories of coding proteins

Class	Description	*Chromohalobacter* 11-W [%]	*Chromohalobacter* 296-RDG [%]	*Chromohalobacter* 48-RD10 [%]	*Halomonas* 11-S5 [%]	*Halomonas* 25-S5 [%]
**Information storage and processing**						
J	Translation, ribosomal structure, and biogenesis	187 [5.85]	184 [5.77]	193 [5.91]	196 [6.14]	194 [5.83]
A	RNA processing and modification	0	0	0	0	0
K	Transcription	265 [8.29]	259 [8.12]	269 [8.23]	223 [6.99]	238 [7.15]
L	Replication, recombination, and repair	129 [4.04]	187 [5.86]	211 [6.46]	178 [5.58]	232 [6.97]
B	Chromatin structure and dynamics	2 [0.06]	1 [0.03]	1 [0.03]	4 [0.13]	4 [0.12]
**Cellular processes and signalling**						
D	Cell cycle control, cell division, chromosome partitioning	50 [1.56]	47 [1.47]	52 [1.59]	48 [1.50]	50 [1.50]
Y	Nuclear structure	0	0	0	0	0
V	Defence mechanisms	34 [1.06]	41 [1.28]	34 [1.04]	50 [1.57]	53 [1.59]
T	Signal transduction mechanisms	135 [4.23]	119 [3.73]	124 [3.80]	156 [4.89]	155 [4.66]
M	Cell wall/membrane/envelope biogenesis	201 [6.29]	227 [7.11]	240 [7.35]	171 [5.36]	176 [5.29]
N	Cell motility	81 [2.54]	80 [2.51]	86 [2.63]	64 [2.01]	62 [1.86]
Z	Cytoskeleton	0	0	0	0	0
W	Extracellular structures	0	0	0	0	0
U	Intracellular trafficking, secretion, and vesicular transport	69 [2.16]	76 [2.38]	77 [2.36]	61 [1.91]	60 [1.80]
O	Posttranslational modification, protein turnover, chaperones	114 [3.57]	108 [3.38]	119 [3.64]	138 [4.33]	137 [4.12]
**Metabolism**						
C	Energy production and conversion	234 [7.32]	203 [6.36]	213 [6.52]	239 [7.49]	250 [7.51]
G	Carbohydrate transport and metabolism	223 [6.98]	224 [7.02]	219 [6.70]	169 [5.30]	171 [5.14]
E	Amino acid transport and metabolism	337 [10.55]	341 [10.69]	330 [10.10]	310 [9.72]	308 [9.26]
F	Nucleotide transport and metabolism	91 [2.85]	88 [2.76]	88 [2.69]	89 [2.79]	92 [2.77]
H	Coenzyme transport and metabolism	148 [4.63]	151 [4.73]	158 [4.84]	149 [4.67]	151 [4.54]
I	Lipid transport and metabolism	119 [3.72]	108 [3.38]	107 [3.28]	135 [4.23]	132 [3.97]
P	Inorganic ion transport and metabolism	231 [7.23]	224 [7.02]	213 [6.52]	233 [7.30]	237 [7.12]
Q	Secondary metabolites biosynthesis, transport, and catabolism	89 [2.79]	73 [2.29]	69 [2.11]	99 [3.10]	101 [3.04]
**Poorly characterized**						
R	General function prediction only	0	0	0	0	0
S	Function unknown	618 [19.34]	614 [19.24]	626 [19.16]	616 [19.31]	646 [19.42]
**All proteins**		3195	3191	3267	3190	3327

**Table 3:** BGCs identified with AntiSMASH in the analysed genomes

Sample name	*Chromohalobacter* 11-W	*Chromohalobacter* 296-RDG	*Chromohalobacter* 48-RD10	*Halomonas* 11-S5	*Halomonas* 25-S5
**AntiSMASH**					
# of BGC	5	7	4	1	1
Arylpolyene	1	1	1	0	0
Betalactone	1	1	0	0	0
Butyrolactone	0	1	0	0	0
Ectoine	1	1	1	1	1
Siderophore	1	1	1	0	0
Phosphonate	0	1	0	0	0
Redox-cofactor	1	1	1	0	0
